# Supramolecular Ring Structures of 7-Methylguanine: A Computational Study of Its Self-assembly and Anion Binding

**DOI:** 10.3390/molecules18010225

**Published:** 2012-12-27

**Authors:** Gábor Paragi, Zoltán Kupihár, Célia Fonseca Guerra, F. Matthias Bickelhaupt, Lajos Kovács

**Affiliations:** 1 Supramolecular and Nanostructured Materials Research Group of the Hungarian Academy of Sciences, University of Szeged, Dóm tér 8, H-6720 Szeged, Hungary; 2 Department of Medical Chemistry, University of Szeged, Dóm tér 8, H-6720 Szeged, Hungary; E-Mails: kupihar.zoltan@med.u-szeged.hu (Z.K.); kovacs.lajos@med.u-szeged.hu (L.K.); 3 Department of Theoretical Chemistry and Amsterdam Center for Multiscale Modeling (ACMM), VU University Amsterdam, De Boelelaan 1083, 1081 HV Amsterdam, The Netherlands; E-Mails: c.fonsecaguerra@vu.nl (C.F.G.); f.m.bickelhaupt@vu.nl (F.M.B.)

**Keywords:** density functional theory calculations, self-assembly, 7-methylguanine, ring structures, anion binding, cooperativity effect, supramolecular structures

## Abstract

The density functional theory calculations of 7-methylguanine clusters revealed that stable ring assemblies can be formed with or without anions in the center position and hexameric clusters are the most stable and most planar ones. The coordination of anions (Cl^−^, Br^−^, NO_3_^−^) stabilizes and thus favors the formation of planar aggregates. We believe that the predicted planar structures stabilized by anions are good models for self-assembly structures formed at solid-liquid or solid-gas interfaces. Comparing the bonding and average H-bond energy to reference ribbon calculations we pointed out the presence of the previously introduced cooperativity effect in circular supramolecular structures of 7-methylguanine.

## 1. Introduction

Higher-order structures based on self-recognition and self-assembly available for simpler molecules are of prime interest in a wide range of research fields starting from structural biology to medicinal chemistry as well as from supramolecular chemistry to nanotechnology. The ability of supramolecular systems to include different molecules, cations and/or anions has long been a flourishing area in this field [1]. The inclusion complexes featuring cations have been pioneered by Pedersen, Cram and Lehn by the introduction of crown complexes and cryptands. Anion complexation studies have been lagging behind those of cations due to some inherent problems (large size, various shapes, solvation effects, narrow pH window, coordinative saturation, *etc*.) [2]. Nonetheless, more and more investigations are directed towards this area of research [3,4,5,6,7].

Nucleic acid self-assembly in the form of various strands (duplexes and triplexes), knots, nucleic acid-metal ion complexes, tetrads and their stacks (quadruplexes) has long been known. The 2D self-assembly of different nucleobase derivatives on surfaces has also been the subject of several studies (e.g., [8,9,10]). It has been demonstrated, e.g., that infinite ribbons and discrete (although interconnected) tetramers of 9-octadecylguanine with cation binding can exist under different conditions and these structures can be interconverted into each other [11]. Further interesting representative of supramolecular structures are the quadruplexes, primarily based on guanine (G) nucleobase in the telomerase region of the chromosome. Although (oligo)nucleotides or other 9-substituted derivatives of guanine are primary targets of several investigations (see e.g., [12]), 7-substituted derivatives have been less investigated. Eukaryotic messenger RNAs are modified at their 5'-ends by addition of a 7-methylguanosine attached by a 5'-5' triphosphate bridge to the first nucleotide of the mRNA chain. This cap structure, m^7^G(5')ppp(5')X (X = any nucleoside), plays a pivotal role in mRNA metabolism, including mRNA transport between the nucleus and the cytoplasm, control of mRNA stability, involving various cap binding proteins [13]. 7-Substituted guanine-containing peptide nucleic acid oligomers are also able to form N7-G*G:C base triplets [14]. In addition, the κ*N*^7^-coordination of guanine residues by platinum complexes has been found to be the primary mode of action of platinum anticancer drugs [15].

We also found that the self-assembly of methylguanines has received little attention so far. Bald *et al.* [16] have found that 2D-self-assembled nanostructures can be formed from guanine and its 1-, 8- and 9-methyl derivatives, respectively. In particular, 1-methylguanine forms windmill structures, 8-methylguanine forms hexagonal networks, guanine and 9-methylguanine form 2D ribbons. The effect of DNA nucleobase methylation is threefold: (i) hydrogen-bonding sites are directly blocked, preventing the formation of particular binding motifs, such as Watson–Crick base pairing; (ii) the strength of intermolecular hydrogen bonds may be modified upon methylation; (iii) an important driving force for 2D self-assembly–cooperative hydrogen bonding–is strongly influenced by the network structure and thus the methylation position.

It is important to emphasize that Bald *et al.* paid special attention to keep free the traditional Watson–Crick and Hoogsten pair connection points in the different methylation patterns of guanine [16]. However, in a brief paper Guille and Clegg [17] obtained small anhydrous guanine crystal with 7*H* tautomeric form and not the usual 9*H* one that is found in monohydrated guanine. The crystal of 7*H*-guanine showed interesting H-bond pattern forming planar sheets, wherein molecules created infinite chains by a triple hydrogen-bonding motif.

A very similar system to Guille and Clegg’s work was investigated by Lippert *et al.* [18]. The self-assembling capacity of protonated 7-methylguanine (Hm^7^G^+^) salts (with different counter ions) and m^7^G–Pt complexes were reported by X-ray experiments. Three counter ions were applied and in case of NO_3_^−^ ion almost perfect parallel sheets were found, similarly to Guille and Clegg’s results. However, the bonding pattern of the ribbons in the sheet differs from those which are based on neutral 7*H*-guanine molecules because of the strong influence by the presence of the anion. Interestingly, none of these works concerned the ring forming capacity of the monomer, however, the tetrameric ring structure in quadruplexes formed by 9-substituted guanines has been very intensively examined as mentioned before [12].

Anion binding capacity of self-assembling nucleobase tetramers has also been less studied. Only a few paper can be found in the literature describing the participation of anions in the stabilization of quadruplexes by anions in lateral positions [19] and according to our knowledge Bickelhaupt *et al.* [20] were the first who have shown theoretically that quartets formed by natural nucleobase can bind halogen anions in the center of their structure.

**Figure 1:**
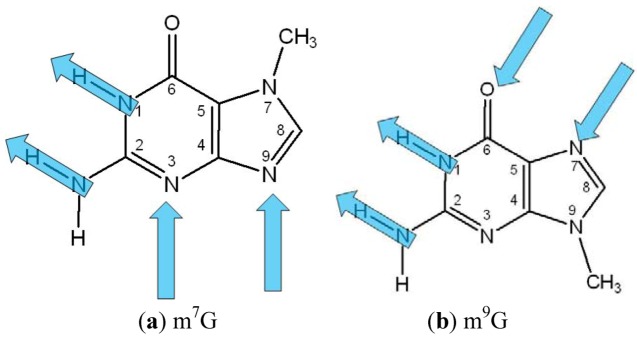
(**a**) The 7-methyl (m^7^G) and (**b**) the 9-methyl (m^9^G) substituted forms of guanine with optimal H-bonding directions in ring formation.

The ring forming ability of 7-methylguanine (m^7^G) has not been examined before, so we investigated herein theoretically the self-assembly capacity of this molecule with special attention to the ring forming possibility on surface. Since planar ribbon structures have been observed with 7*H*-guanine, we apply the ribbon motif as a reference system. In Figure 1 we present m^7^G and 9-methylguanine (m^9^G) for comparison, where arrows indicate the direction of H-bonds in self-assembled ring structures.

It is clear that the angle between the optimal donor and acceptor directions is quite different in the two cases. For m^7^G it is definitely larger than 90°, while in m^9^G assemblies it is nearly perpendicular. Consequently, four molecules can form a closed planar ring motif in the latter situation (which is the building block of the previously mentioned quadruplex structures), while in the former one, five or six units are necessary to reach a nearly planar ring structure. To check this assumption 4-, 5- and 6-membered ring structures of m^7^G were optimized with high level quantum chemical method. For comparison, we also performed plane-restrained ribbon fragment calculations of m^7^G built up from 4, 5, 6 and 7 units. Examination of ring structure with more than 4 units is based on the result of isoguanine (6-amino-1,7-dihydropurin-2-one) investigations where this natural but non-standard base has been found to form not only quartets but also pentamers, depending on the cation identity [21,22,23].

## 2. Theoretical Methods

### 2.1. Computational Details

All calculations were performed using density functional theory (DFT) as implemented in the Amsterdam Density Functional (ADF) program developed by Baerends and others [24] and the QUantum-regions Interconnected by Local Descriptions (QUILD) program by Swart and Bickelhaupt [25,26]. The calculations were performed at BLYP-D/TZ2P level of theory for tetramers, and the final geometry was determined with 10^−5^ and 10^−6^ accuracy for the gradient and the energy, respectively. The BLYP functional was augmented with dispersion correction according to Grimme’s suggestion (BLYP-D calculation) [27,28]. In this approach, the BLYP density functional is augmented with an empirical correction term for long-range dispersion effects, described by a sum of damped interatomic potentials of the form C_6_R^−6^ added to the usual DFT energy. This approach has been shown to yield excellent structures and energies for multiply-hydrogen bonded DNA-base oligomers [20,29].

### 2.2. Bonding Analyses

The overall bond energy ΔE_bond_ is made up of two major components [Equation (1)]:

ΔE_bond_ = ΔE_prep_ + ΔE_int_ (1)

In this formula, the preparation energy ΔE_prep_ is the amount of energy required to deform the separate monomer bases from their equilibrium structure to the geometry that they acquire in the ribbon. The interaction energy ΔE_int_ corresponds to the actual energy change when the chosen geometrically deformed constituents are combined to form the ring or ribbon complexes (E_complex_ − ΣE_constituents_). E.g., when anion binding is calculated then the two constituents are the separated ion and the ion-free ring, and the deformation energy of the ring is related to the optimized ion-free ring structure. In the meantime, the bonding energy of the same complex is related to the separated six m^7^G units and the detached ion. It is worth to note that the sum of preparation energies is one order of magnitude smaller than the interaction energy and usually 1–2 kcal/mol for a single m^7^G unit.

## 3. Results and Discussion

### 3.1. Systems without Ions

**Figure 2:**
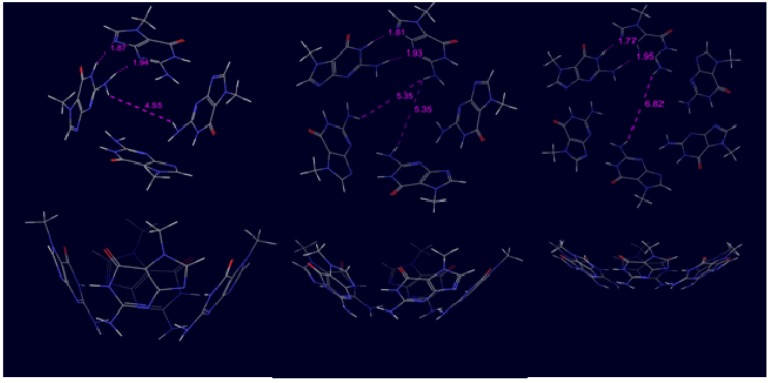
Calculated geometries of 4-, 5- and 6-membered ring conformations of m^7^G (top and side view) with selected distances (in Å).

The optimized ring geometries with selected distances can be found in Figure 2 and the related bonding energies are shown in Table 1 as well as the results of the reference ribbon calculations.

**Table 1:** Bonding energies and average H-bond energies (in kcal/mol) of the 4-, 5- and 6-membered m^7^G rings (with and without C_s_ symmetry) and the planar ribbon structures with 4, 5, 6 and 7 units. The number of H-bonds in the concerned structure is also shown.

	Tetramer			Pentamer			Hexamer			Heptamer		
	Energy (kcal/mol)		H-bonds	Energy (kcal/mol)		H-bonds	Energy (kcal/mol)		H-bonds	Energy (kcal/mol)		H-bonds
	Bonding	Average H-bond		Bonding	Average H-bond		Bonding	Average H-bond		Bonding	Average H-bond	
Ring **without** C_s_ sym.	−71.9	−9.0	8	−96.7	−9.7	10	−120.4	−10.0	12	-		
Ring **with** C_s_ sym.	−53.3	−6.7		−76.6	−7.7		−114.0	−9.5				
Ribbon **with** C_s_ sym.	−78.1	−8.7	9	−102.1	−8.5	12	−130.7	−8.7	15	−156.3	−8.7	18

The optimized geometries show clearly that the ring assembly tends to be planar with increasing number of ring members which is also corroborated by comparing bonding energies of planar and non-planar structures. Namely, the bonding energy difference between the C_s_ constrained and freely optimized geometries is 6.4 kcal/mol in case of the hexameric ring while the same difference is 18.7 kcal/mol and 20.1 kcal/mol in tetrameric and pentameric cases, respectively. These numbers also demonstrate that the hexameric ring arrangement can be favorable therefore we applied this unit number in anion-containing calculations. 

**Figure 3:**
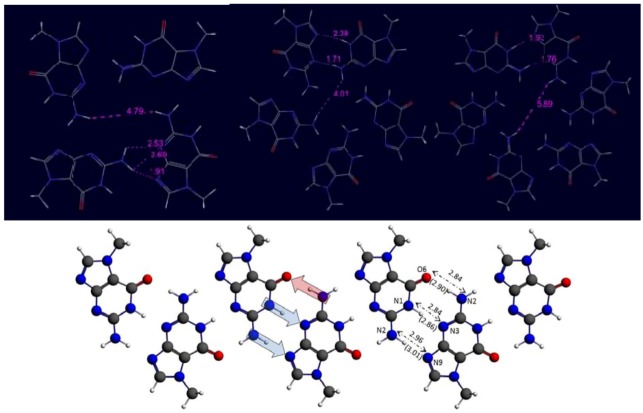
Optimized planar ring and ribbon structures of m^7^G with selected distances (in Å). The numbers in parenthesis show the corresponding distances in 7*H*-guanine crystal [17]. The hollow arrows represent the D-H…A hydrogen bonding pattern.

In Figure 3 the optimized planar ring conformations are presented together with the seven units-long ribbon conformation. The appropriateness of the chosen calculation method can be demonstrated by comparing theoretical and experimental results. We calculated the length of the translation symmetry vector in the optimized ribbon structure and compared to 7*H*-guanine crystal data. In the former case we get 9.8 Å while in the latter one the result was 9.7 Å. Moreover, comparing the heavy atom distances involved in H-bonding in ribbon we have obtained 2.84 Å for N1-H1…N3, 2.96 Å for N2-H2…N9 and 2.84 Å for N2-H2…O6 (see Figure 3). The corresponding values found in crystals are 2.86 Å, 3.01 Å and 2.90 Å, respectively [17], which are in good accordance with the computed ones. These data confirm the previous analyses which demonstrate that this level of theory yields excellent structures and energies for multiply-hydrogen bonded DNA-base oligomers [20,29].

Comparing the ring and ribbon bonding energies with the same number of units one can see that even in the tetrameric cases the two extra (ring-closing) H-bonds cannot compensate the bonding energy of ribbon structure formed by three H-bonds. However, the average H-bond energies demonstrate an interesting difference between ribbon and ring structures. Namely, while the average H-bond energy of ribbons does not change with the increasing number of units, in the ring situations the H-bond energy displays a definitely increasing tendency, independently from the presence or absence of C_s_ symmetry restraint.

The theoretical explanation of this fact is based on our previous work [30], where tetrameric xanthine and guanine structures were examined. We found in that paper that extra stability can occur only in the presence of a special H-bond arrangement. Namely, the donor-acceptor direction of adjacent H-bonds must be the same to reach extra stability. In Figure 2 and Figure 3 one can easily check that this requirement is fulfilled in ring structures, but in ribbon arrangement the H-bond between the oxo and the amino groups is oppositely directed compared to the other two H-bonds. It is important to emphasize that the present systems display cooperative effect even at non-planar calculations as it is shown by increasing average H-bond energies. Considering planar systems we pointed out in ref. [30] that cooperativity is primarily related to the donor–acceptor orbital interactions in the σ-electron system, and not from the strengthening caused by resonance in the π-electron system. However, the π-electron system in the present structures is different from the one which can be found in a perfectly planar geometry. Moreover, we would like to emphasize that according to the ribbon calculations one oppositely directed H-bond can prevent the appearance of cooperativity. This fact is shown by the present structures where the principal difference between ring and ribbon structure is the presence or the absence of the third H-bond formation between the oxo and the amino groups. 

Finally, we would like to note that further investigations based on different energy decomposition methods can be useful to refine the description of the cooperative character. The application of the Ziegler-Rauk method [31,32], as well as the decomposition of energy components according to paper [33] into two-body, three-body, *etc.* terms can be a useful method. However, in the present paper we primarily focus on the possible formation and ion binding capacity of this systems, therefore these more detailed analyses are beyond the scope of this work.

### 3.2. Hexameric Ring with Selected Anions

**Figure 4:**
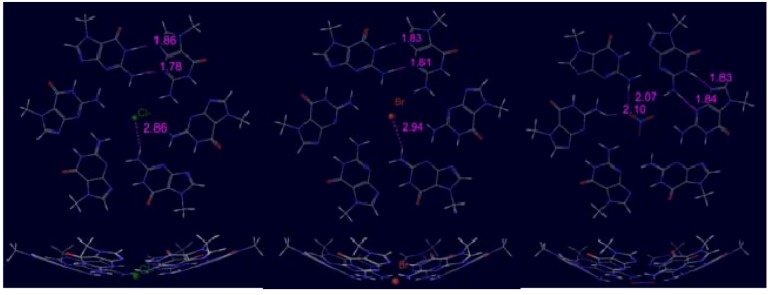
Optimum geometry of m^7^G hexamer with selected (Cl^−^, Br^−^, NO_3_^−^) anions without C_s_ symmetry.

The most interesting property of the new structures is that, in contrast to the usual G tetrad, they can bind anions in the central position and not just monoatomic anions (Cl^−^ or Br^−^) but oxoanions (e.g., CO_3_^2−^ or NO_3_^−^) as well. This kind of binding was suggested theoretically for Cl^−^ at the first time by Bickelhaupt *et al.* in reference [20] where adenine tetramer could bind the anion in the center of the structure. In Figure 4 we present unconstrained hexameric complexes where selected anions have been investigated in the central position. Bonding energies and anion binding energies are collected in Table 2 for bent and C_s_ constrained planar calculations.

**Table 2:** Bonding energies and ion bonding energies (in kcal/mol) of hexameric m^7^G ring in the presence of selected anions (Cl^−^, Br^−^ and NO_3_^−^). Optimizations were carried out with and without C_s_ symmetry.

Hexameric ring with anions	Cl^−^		Br^−^		NO_3_^−^	
	Bonding energy (in kcal/mol)	Ion bonding energy	Bonding energy (in kcal/mol)	Ion bonding energy	Bonding energy (in kcal/mol)	Ion bonding energy
without C_s_ sym.	−170.8	−50.3	−168.3	−47.6	−177.0	−56.3
with C_s_ sym.	−170.3	−50.1	−167.7	−47.6	−174.3	−54.1

Comparing the bonding energies of free systems to the plane-constrained one we found that in the presence of a central anion the energy difference between bent and planar structures is much smaller than in the absence of the anion. For monoatomic cases it is less than 1 kcal/mol and even for the NO_3_^−^ ion it is only 2.7 kcal/mol. The rest of the change comes from the ion bonding energy difference between planar and bent geometries, more precisely from the deformation energy.

One can conclude from the results that these facts demonstrate the possible coordination role of anions in the formation of ring structures. In other words, hexameric ring formation at solid-liquid or solid-gas interfaces is more probable in the presence of anions.

It is worth to note that if one substracts the ion bonding energy from the bonding energy the results were always between −120.1 kcal/mol and −120.7 kcal/mol, independently from the chosen anion or the presence/absence of C_s_ symmetry. Taking into account that the bonding energy of the freely optimized hexameric ring is −120.4 kcal/mol, while the one with C_s_ constrain is −114.0 kcal/mol, we can interpret this result in such a way that the anion drives the ring structure to the most suitable state.

## 4. Conclusions

We have demonstrated that density functional theory calculations on m^7^G forecast the formation of ring clusters with or without anion in the center position. The coordination of anions stabilizes and thus favors the formation of planar aggregates. Comparing the calculation results of C_s_ restrained m^7^G ribbon structure to 7*H*-guanine crystal geometry we found an excellent accordance between the experimental and the calculated geometries. Based on this good agreement, we believe that the predicted planar structures stabilized by anions (Cl^−^, Br^−^, NO_3_^−^) are good models for self-assembly structures formed at solid-liquid or solid-gas interfaces.

The bonding and average H-bond energy differences between ring or ribbon structures can be interpreted very well by presence or absence of the previously introduced cooperativity effect. In the ring structures with their unidirectional hydrogen bonds, there is a cooperativity effect that increases with the number of monomers, in line with and explained in our previous work [30]. Such a cooperativity is not present in the ribbon structures in which oppositely directed H-bonds quench the cooperative effect. 
